# Editorial: Positive Psychological Assessments: Modern Approaches, Methodologies, Models and Guidelines: Current perspectives

**DOI:** 10.3389/fpsyg.2022.1020653

**Published:** 2022-09-26

**Authors:** Arianna Costantini, Leon T. De Beer, Peter M. ten Klooster, Marielle A. J. Zondervan-Zwijnenburg, Maria Vera, Llewellyn Ellardus van Zyl

**Affiliations:** ^1^Department of Psychology and Cognitive Science, University of Trento, Trento, Italy; ^2^WorkWell Research Unit, North-West University, Potchefstroom, South Africa; ^3^Department of Psychology, Health and Technology, University of Twente, Enschede, Netherlands; ^4^Department of Methodology and Statistics, Utrecht University, Utrecht, Netherlands; ^5^Social Psychology Department, Universidad Pablo de Olavide, Sevilla, Spain; ^6^Department of Industrial Engineering and Innovation Sciences, University of Eindhoven, Eindhoven, Netherlands; ^7^Optentia Research Unit, North-West University, Vanderbijlpark, South Africa; ^8^Department of Human Resource Management, University of Twente, Enschede, Netherlands; ^9^Department of Social Psychology, Institut für Psychologie, Goethe University, Frankfurt am Main, Germany

**Keywords:** applied positive psychology, mental health, positive psychology, positive psychological assessment, positive psychology coaching, scale development, wellbeing, wellbeing assessment

## Introduction

Sparked by evidence showing that positive psychological approaches and practices not only foster flourishing but also help to reduce mental illness, maintain mental health, and strengthen one's psychological resources and capacities (Waters et al., 2022), positive psychology interventions and coaching have emerged as popular approaches for practitioners interested in the development and wellbeing of people (Lomas, 2020; Moskowitz et al., 2021; Richter et al., 2021). Indeed, bourgeoning evidence for the social, behavioral, and physical health benefits of positive psychology constructs (Donaldson et al., 2021; Moskowitz et al., 2021) has led to an increasing influence of positive psychology underpinning practice (Green and Palmer, 2019), with significant growth of the use of positive psychological assessment measures (PPAMs).

Despite such popularity, research has also highlighted the shortcomings of existing PPAMs (e.g., Wong and Roy, 2017; van Zyl and Rothmann, 2022), with important implications for a valid and reliable assessment of the effectiveness of positive psychology practices as well as the advancement of our understanding of wellbeing through the development and conceptualization of new positive psychological constructs (Gruman et al., 2018; van Zyl and Rothmann, 2022; Van Zyl and Salanova, 2022). For example, it has been argued that the broad category of wellbeing, encompassing independent and separable components (Diener, 1984; Ng et al., 2021), is inconsistently operationalized across studies, making it difficult to determine whether positive psychology interventions have stronger effects on particular aspects of wellbeing compared to others (Moskowitz et al., 2021). Similarly, across different studies, diverse positive constructs can be found grouped together, for example, combining positive emotions with outcomes (e.g., meaning, purpose, life satisfaction; Sin and Lyubomirsky, 2009; Chakhssi et al., 2018) or with other cognitive and affective appraisals of one's life as a whole (Bolier et al., 2013; Hendriks et al., 2019; Moskowitz et al., 2021).

Given the key role of PPAMs in advancing the science and practice of positive psychology (van Zyl and Rothmann, 2022), this Research Topic specifically focused on collecting evidence and informed proposals of modern approaches, methodologies, models, and guidelines for PPAMs.

## Structure and contribution of the Research Topic

The contributions included in this Research Topic are summarized in Table 1 and presented below. In summary, responding to our call for more thorough evidence on the validity and reliability of both newly developed and translated popular PPAMs, nine manuscripts included in this Research Topic focused on investigating the psychometric properties of different PPAMs using a variety of modern statistical modeling techniques and across different cultural contexts. Moreover, two contributions used survey data to provide an example of best practice guidelines and investigate the properties of positive psychological constructs using modern approaches. Finally, one contribution presented a systematic review of observational PPAMs developed to assess momentary wellbeing in people living with dementia.

**Table 1:** Characteristics of the studies included in the Research Topic.

**References**	**Construct**	**PPAM**	**Dimensions & *N* of items**	**Participants**	**Country**	**Language PPAM used in the research**
***Scale development and validation***
Bauman and Ruch	Fulfillment in life	Fulfilled Life Scale (FLS)	Fulfilled Life Cognitive Experience (24) Fulfilled Life Affective Experience (8)	Sample 1: 282 adults (50–93 yo) Sample 2: 406 adults (40–85 yo) Sample 3: 39 adults (41–89 yo)	German-speaking countries	German
Carmona-Halty et al.	Flourishing	Flourishing Scale (FS) adapted to the educational setting	Flourishing (8)	1,348 students (13–18 yo)	Chile	Spanish
Chen et al.	Life crafting	Life Crafting Scale (LCS)	Cognitive Crafting (3) Seeking Social Support (3) Seeking Challenges (3)	Sample 1: 331 employees Sample 2: 362 employees	United Kingdom, Portugal, Poland, The Netherlands	English
Cromhout et al.	Eudaimonic wellbeing	Questionnaire for Eudaimonic WellBeing (QEWB)	Sense of Purpose (7) Purposeful Personal Expressiveness (9) Effortful Engagement (5)	Sample 1: 326 univ students Sample 2: 478 univ students Sample 3: 260 univ students Sample 4: 262 adults	South Africa	Sample 1, 4: English Sample 2: Afrikaans Sample 3: Setswana
Espejo et al.	Satisfaction with life	Satisfaction with Life Scale (SWLS)	Satisfaction with Life (5)	1,255 adults	Colombia	Spanish
Guitard et al.	Satisfaction with life	Temporal Satisfaction with Life Scale (TSWLS)	Past Life Satisfaction (4) Present Life Satisfaction (4) Future Life Satisfaction (4)	3,982 Eng-speakers (over 16 yo) 2,930 non-Eng speakers (over 16 yo)	Worldwide	English Hungarian Spanish Finnish Slovene Czech Chinese
van Zyl et al.	Strengths use	Strengths Use Scale (SUS)	Affinity for Strengths (6) Strengths Use Behaviors (8)	360 univ students	The Netherlands	Dutch
Youssef-Morgan et al.	Work gratitude	Work Gratitude Scale (WGS)	Grateful appraisals (3) Gratitude toward others (4) Intentional attitude of gratitude (3)	625 employees	USA	English
Zábó et al.	Mental health	Mental Health Test (MHT)	Wellbeing (3) Savoring (3) Creative and Executive Efficiency (5) Self-Regulation (3) Resilience (4)	Sample 1: 1,736 adults Sample 2: 1,083 adults	Hungary	Hungarian
***Guidelines and Survey Design Papers***
van Zyl and ten Klooster	Mental health	Mental Health Continuum-Short Form (MHC-SF)	Emotional wellbeing (3) Social wellbeing (5) Psychological wellbeing (6)	1,804 adults	The Netherlands	Dutch
Ratchford et al.	Mindsets across domains	Implicit Theories of Morality and Intelligence Scale	Morality mindset (3) Ability mindset (3)	618 adolescents (15–19 yo)	United States	English
***Systematic review***
Madsø et al.	Momentary wellbeing	Observational instruments assessing momentary wellbeing		People with dementia		

The dimensions and number of items reported are based on the results of the studies. yo, years old; univ, university; Eng, English.

### Scale development and validation

Bauman and Ruch presented a study on the development and validation of the Fulfilled Life Scale, capturing people's experience of a fulfilled life. Using data from three different German-speaking samples (development sample *n* = 282; replication sample *n* = 406; selected exemplar participants *n* = 39), they identified three optimal factors across cognitive and affective experiences of a fulfilled life, labeled *unfolded self and life*, the *worthwhile life*, and *positive impact and legacy*. Cognitive and affective fulfillment incrementally predicted a global rating of a fulfilled life and mental wellbeing, even after controlling for subjective and eudaimonic wellbeing.

Carmona-Halty et al. presented an adaptation of the Flourishing Scale (FS) to a Chilean high school context and provided evidence of its validity. Using a cross-sectional sample of 1,348 students from three different schools in Chile, they showed that their adapted version of the FS is invariant across genders and is positively related with study–related positive feelings (i.e., happiness, pleasure, and satisfaction) and negatively related with study–related negative feelings (i.e., sadness, displeasure, and anger).

Focusing on behaviors aimed to better align life goals, personal needs, values, and capabilities, Chen et al. proposed a conceptualization of life crafting and developed, validated, and evaluated a measure of overall life crafting, the Life Crafting Scale (LCS). Using a mixed-method, multi-study research design, in the first qualitative phase, they created a pool of items; then, in Study 1, involving 331 English-speaking employees, they found support for a three-factor structure encompassing *cognitive crafting, seeking social support*, and *seeking challenges*. In Study 2, involving 362 employees in the Netherlands, the factorial structure of the scale was confirmed, and the LCS showed to be a reliable tool, partially invariant across genders, and positively associated with meaning in life, mental health, and work engagement, while negatively related to job burnout.

Cromhout et al. zoomed into the construct of eudaimonic wellbeing and used various analytical models, including CFA, bifactor CFA, Exploratory Structural Equation Modeling (ESEM), and bifactor ESEM, to investigate the dimensionality of the Questionnaire for Eudaimonic WellBeing (QEWB) in four culturally diverse South African samples, including three student samples (English, *n* = 326; Afrikaans, *n* = 478; and Setswana, *n* = 260) and one multicultural adult sample (*n* = 262). Their results showed that eudaimonic wellbeing is multidimensional but, at the same time, represents an overarching higher-order construct. Moreover, they found configural invariance across the different languages in which the QEWB was administered, but also that the QEWB shows differential psychometric properties across different age groups and developmental phases.

Espejo et al. focused on the Satisfaction with Life Scale (SWLS) with five response options and investigated its psychometric properties in a Colombian sample of 1,255 participants. Their results showed that the SWLS, in its Spanish version used in Colombia, is a reliable and valid tool displaying excellent psychometric properties and invariance across genders and age groups. Also, as expected, it correlates significantly with life satisfaction, flourishing, positive and negative affect, optimism, and pessimism.

Guitard et al. provided a thorough investigation of the Temporal Satisfaction with Life Scale (TSWLS) structure and number of optimal items. Based on a large international and multicultural sample (*n* = 6912), their findings showed that a 12-item version of the scale was optimal compared to the original 15-item one, and it was equivalent and valid across English speakers in different geographic regions of the world, including Oceania, North America, Europe, and Asia. Also, their results showed that six different translations of the TSWLS function in similar ways, yet some differences exist in item functioning across cultures. All three subscales of the TSWLS, that is, past, present, and future, displayed positive correlations with aspects of wellbeing (strengths use and knowledge, subjective happiness, gratitude, hope, and the presence of meaning in life) and negative ones with aspects of ill-being (search for meaning in life, rumination, depression), as expected.

Van Zyl et al. investigated the psychometric properties, longitudinal invariance, and criterion validity of the Strengths Use Scale (SUS) within 360 students in the Netherlands. Their results showed that the SUS comprises two first-order factors, namely *affinity for strengths* and *strengths use behaviors*. This factorial structure showed to be consistent across time, and longitudinal evidence showed that strengths use remained stable over time. Moreover, strengths use predicted study engagement assessed 3 months after, providing evidence of the criterion validity of the SUS.

Youssef-Morgan et al. introduced the new construct of work gratitude, defined as “the intentional choice to engage in positive appraisals and feelings of thankfulness and appreciation toward the characteristics, situations, and people currently present in the work context,” and presented a new instrument, the Work Gratitude Scale (WGS), to assess it. Using cross-sectional data from 625 employees from a school district in the United States, they found support for the validity of a second-order model of work gratitude with three underlying dimensions: *grateful appraisals, gratitude toward others*, and *intentional attitude of gratitude*. The WGS showed to be a valid and reliable instrument, useful to spark research on how to promote grateful appraisals, gratitude toward others, and intentional attitudes of gratitude in employees.

Zabo et al. presented a new five-scale mental health test, the Mental Health Test (MHT), that operationalizes a set of indicators of a newly introduced concept of positive mental health. Based on cross-sectional self-reported data collected in Hungary, they found support for a five-factor structure with 17 items (*n* = 1736), which was confirmed in a separate sample (*n* = 1083). The MHT maps aspects of wellbeing, savoring, creative and executive efficiency, self-regulation, and resilience. Results showed that the MHT displayed a high level of internal consistency, and correlations confirmed the content validity of the subscales with established measures of psychological wellbeing. Moreover, test-retest reliability was confirmed by longitudinal data collected after 2 weeks and again after 11 months. Overall, their results showed that the MHT could be considered a new reliable, valid measurement tool to assess several aspects of mental health.

### Guidelines and survey design papers

Using data collected on the Mental Health Continuum-Short Form (MHC-SF) as an illustrative example, van Zyl and ten Klooster provided a practical tutorial on how to use Exploratory Structural Equation Modeling (ESEM) with a convenient online tool for M*plus*. In their paper, they illustrated the applicability of the ESEM as an alternative to traditional CFA approaches and provided an overview of ESEM and structured guidelines on how to use and apply ESEM models, including a step-by-step guide to producing ESEM syntax to be used with M*plus*. Contributing to the literature on mental health assessment, they further showed that when measuring mental health with the MHC-SF, an approach that accounts for a bifactor ESEM model should be preferred to CFA models.

Focusing on mindsets across different domains, Ratchford et al. contributed evidence to the debate about domain specificity and generality of mindset while exploring how cohesion and divergence across moral and ability mindsets affect self-system, self-regulatory strategies, and wellbeing outcomes in a sample of 618 adolescents in metropolitan southern California. To assess congruence and discrepancies, they used response surface analysis to consider the within-person effects of domain specificity across various outcomes. Their findings showed that overall congruency between moral and ability mindsets did not relate significantly with any of the wellbeing outcomes considered, suggesting that ability and moral mindsets are distinct qualities for which congruence is not relevant for wellbeing. Hence, by showing that mindsets display high levels of domain specificity, this study offers implications for the assessment of mindsets as characteristic adaptations, suggesting that different mindsets should therefore be assessed and accounted for independently in future survey designs.

### Systematic review paper

Madsø et al. presented a systematic review of 36 articles describing 22 observational instruments assessing momentary wellbeing in people with dementia. The instruments included in the review mapped three categories: observations of emotions, observations of positive behavioral expressions, and observations of engagement. Their analysis included risk of bias at the study level and assessment of measurement properties at the instrument level (content validity, construct validity, structural validity, internal consistency, measurement invariance, cross-cultural validity, measurement error, and inter-rater/intra-rater/test-retest reliability and responsiveness). Results showed that among the instruments included in the review, 11 were supported by high-quality evidence for content validity, while the presence of high-quality evidence of other central psychometric aspects was sparse. However, several instruments have the potential to meet such quality criteria if further investigated.

## Conclusion

While much research in positive psychology is primarily situated within a positivistic paradigm and adopts quantitative designs (Rich, 2017; Gruman et al., 2018; Lomas et al., 2021), there is still a need for a more robust understanding of the properties of—both widespread and newly developed—PPAMs, so as to enhance the credibility of the discipline and our knowledge and impact of positive psychology (van Zyl and Rothmann, 2022). In an effort to address these challenges, this Research Topic provided a collection of contributions on assessment tools and operationalizations of positive psychology constructs, allowing us to gauge evidence regarding different approaches and instruments needed to understand the conditions and processes that foster optimal functioning and flourishing in people, groups, and institutions.

## Author contributions

AC drafted the first version of the editorial. LvZ edited the manuscript. All authors provided conceptual input and approved the final draft.

## Conflict of interest

The authors declare that the research was conducted in the absence of any commercial or financial relationships that could be construed as a potential conflict of interest.

## Publisher's note

All claims expressed in this article are solely those of the authors and do not necessarily represent those of their affiliated organizations, or those of the publisher, the editors and the reviewers. Any product that may be evaluated in this article, or claim that may be made by its manufacturer, is not guaranteed or endorsed by the publisher.
